# Endobronchial Therapy With Gentamicin and Dexamethasone After Airway Clearance by Bronchoscopy in Exacerbation of Non-Cystic Fibrosis Bronchiectasis: A Real-World Observational Study

**DOI:** 10.3389/fphar.2021.773241

**Published:** 2021-11-11

**Authors:** Qiuhong Li, Beijie Huang, Hongyan Gu, Ying Zhou, Xizheng Shan, Siming Meng, Meng Qin, Jingyun Shi, Yanan Chen, Huiping Li

**Affiliations:** ^1^ Department of Respiratory and Critical Care Medicine, Shanghai Pulmonary Hospital, School of Medicine, Tongji University, Shanghai, China; ^2^ Department of Respiratory Medicine, Yangpu Hospital, School of Medicine, Tongji University, Shanghai, China; ^3^ Department of Respiratory Medicine, The Sixth People’s Hospital of Nantong, Nantong, China; ^4^ Department of Radiology, Shanghai Pulmonary Hospital, School of Medicine, Tongji University, Shanghai, China

**Keywords:** bronchiectasis, exacerbation, bronchoscopy, gentamicin, dexamethasone

## Abstract

**Background:** The exacerbation of non-cystic fibrosis bronchiectasis (NCFB) may lead to poor prognosis. The objective of this study was to retrospectively analyze the clinical efficacy and safety of endobronchial therapy with gentamicin and dexamethasone after airway clearance by bronchoscopy in the exacerbation of NCFB.

**Methods:** We retrospectively reviewed 2,156 patients with NCFB between January 2015 and June 2016 and 367 consecutive patients with exacerbation of bronchiectasis who had complete data and underwent airway clearance (AC) by bronchoscopy. The final cohort included 181 cases of intratracheal instillation with gentamicin and dexamethasone after AC (a group with airway drugs named the drug group) and 186 cases of AC only (a group without airway drugs named the control group). The last follow-up was on June 30, 2017.

**Results:** The total cough score and the total symptom score in the drug group were improved compared to those in the control group during 3 months after discharge (*p* < 0.001). Re-examination of chest HRCT within 4–6 months after discharge revealed that the improvements of peribronchial thickening, the extent of mucous plugging, and the Bhalla score were all significantly improved in the drug group. Moreover, the re-exacerbations in the drug group were significantly decreased within 1 year after discharge. Univariate analysis showed a highly significant prolongation of the time to first re-exacerbation in bronchiectasis due to treatment with airway drugs compared with that of the control group. Multivariate Cox regression analysis showed that the risk of first re-exacerbation in the drug group decreased by 29.7% compared with that of the control group.

**Conclusion**: Endobronchial therapy with gentamicin and dexamethasone after AC by bronchoscopy is a safe and effective method for treating NCFB.

**Clinical Trial Registration**: http://www.chictr.org.cn, identifier ChiCTR1900022247.

## Introduction

Non-cystic fibrosis bronchiectasis (NCFB) is a chronic suppurative and inflammatory lung disease of diverse etiology characterized by pathological and irreversible dilatation of the bronchial tree (Polverino et al., 2017). The impairment of the mucociliary clearance, which results from chronic airway inflammation, may cause long-term colonization or recurrent infection of bacteria, especially *Pseudomonas aeruginosa (PA)*, while bacterial colonization and recurrent infection can aggravate airway inflammation (Flume et al., 2018). Sputum retention caused by the impairment of mucociliary clearance can result in mucous plugs, which in turn contribute to airflow obstruction and dyspnea (McShane et al., 2013). Clinically, the major manifestations of NCFB are chronic cough with purulent sputum, dyspnea, and fatigue that may diminish the patients’ quality of life (King et al., 2006; Martínez-García et al., 2006). The frequency of exacerbation and the decline in lung function may lead to poor prognosis in NCFB (Loebinger et al., 2006). The mean frequency of exacerbations per NCFB patient per year was 1.8 (SD, 1.4), with a hospitalization rate from 26.6 to 40.4% (Chalmers et al., 2014). The expected mortality rate in patients with NCFB is more than twice as high as that in healthy people (Loebinger et al., 2006).

The purpose of NCFB management is to reduce exacerbation, prevent complications, and improve the quality of life (Polverino et al., 2017). Long-term nebulized gentamicin can reduce the concentration of bacteria in the airways, decrease sputum production, attenuate lung function decline, and reduce acute pulmonary exacerbations without nephrotoxicity or ototoxicity (Murray et al., 2011). Yet, some patients may have bronchospasm, dyspnea, and chest pain and may not tolerate long-term nebulized antibiotics (Couch, 2001; Haworth et al., 2019). Dexamethasone is one of the most common glucocorticoids which can inhibit the expression levels of inflammatory factors in the airway and reduce the secretion of airway mucus (Chan et al., 2008). Nimmo et al. investigated the pulmonary deposition of dexamethasone after intratracheal instillation by using radiolabeled dexamethasone in the Survanta mixture to rats (Nimmo et al., 2002). They found that the instilled dexamethasone was well-distributed throughout all four lobes of the lungs. A similar distribution of dexamethasone was observed when using saline as the vehicle. Topical administration could also reduce the systemic side effects. Based on these research studies, we have found that endobronchial therapy with gentamicin and dexamethasone after airway clearance (AC) by bronchoscopy in the exacerbation of NCFB could alleviate the severity of bronchiectasis and reduce the occurrence of exacerbations without any obvious side effects, after many years of exploration in clinical practice. In the current study, we retrospectively reviewed the diagnosis and treatment of 367 patients with exacerbation of NCFB in order to preliminarily evaluate the efficacy and safety of this method.

## Materials and Methods

### Patients

Data from patients with exacerbation of NCFB who underwent AC by bronchoscopy were retrospectively collected from inpatients at the Shanghai Pulmonary Hospital (Shanghai, China) between January 1, 2015 and June 30, 2016. The last follow-up was performed on June 30, 2017.

The inclusion criteria were as follows: patients with NCFB confirmed by chest high-resolution computed tomography (HRCT); patients needing antibiotic treatment at the hospital due to exacerbation (Pasteur et al., 2010); and patients who underwent AC by bronchoscopy at the hospital. Exclusion criteria were as follows: the presence of cystic pulmonary fibrosis; patients with active pulmonary tuberculosis; those awaiting surgery; patients who underwent interventional therapy for hemoptysis; those with allergic bronchopulmonary aspergillosis (ABPA); and patients who did not undergo bronchoscopy for different reasons or combined with lung cancer.

In the drug group (a group treated with airway drugs), the mixture of 2 ml of 0.9%-physiological saline was pre-heated to 37°C (Otsuka Pharmaceutical Co. Ltd.) along with 2 ml gentamicin sulfate (80000U, Yichang Renfu Pharmaceutical Co. LTD) and 1 ml dexamethasone sodium phosphate injection was administered (5 mg, Guangzhou Baiyunshan Tianxin Pharmaceutical Co. Ltd.). Topical intrabronchial therapy was administered by using the abovementioned saline, gentamicin, and dexamethasone after AC by bronchoscopy. The patients from the control group (the group treated without airway drugs) had recently undergone AC by bronchoscopy.

### Interventions

All patients received routine treatment, such as anti-inflammation and symptomatic treatment, at the hospital, and they all signed the informed consent form before bronchoscopy. After the airway mucus was removed, the bronchial tubes in which the NCFB was located were irrigated by 10–20 ml of 0.9% physiologic saline at 37°C, after which the bronchoalveolar lavage fluid was retrieved for testing. According to the amount of mucus in the airway, appropriate irrigation can be carried out repeatedly. There were no other drugs in the bronchus of the control group. The aforementioned mixture of gentamicin, dexamethasone, and saline was infused according to the region of the lesion in the drug group, for example, in the bifurcation region of the bronchus of localized bronchiectasis and in the bifurcation region of the upper (including the lingular segment) or lower lobe (including right middle lobe) of the bronchus on one side or both sides depending on unilateral or bilateral diffuse NCFB. An ECG monitor instrument was used during and after the operation to observe the presence of adverse events.

### Assessments

We collected the hospitalization data (as baseline data) of patients during the exacerbation of NCFB, including age, gender, smoking history, body mass index (BMI), duration of NCFB, past medical history and complications, clinical symptoms, blood routine test, arterial blood gas, microbial culture results of sputum and alveolar lavage fluid, pulmonary function, and chest HRCT. The follow-up data included the clinical symptoms present at 3 months after discharge, the chest HRCT within 4–6 months after discharge, the numbers of exacerbation within 12 months after discharge, and the time taken to the first re-exacerbation and the use of antibiotics after discharge. The clinical symptom score scale of NCFB was developed according to the domestic and foreign literatures (Torrego et al., 2006; Quittner et al., 2015; Bai et al., 2014) (Supplementary Table 1). The chest HRCT was independently reviewed by two experienced radiologists, and the severity of bronchiectasis was graded by using the Bhalla scoring system (Bhalla et al., 1991; Oikonomou et al., 2008) (Supplementary Table 2). The cumulative months of oral and intravenous antibiotics were recorded within 1 year after discharge.

### Statistical Analysis

The measurement data were all expressed by mean ± SD (standard deviation), and the counting data were expressed by rate (%) or composition rate (%). Student’s *t*-test was used for comparison of the normally distributed continuous data, and the Mann–Whitney *U* test was used for non-normally distributed data. The differences of categorical variables were assessed by the chi-square test or Fisher’s exact test. A two-tailed *p*-value less than 0.05 was considered statistically significant. The Kaplan–Meier curves were used to evaluate differences between patients in the drug and control groups for the time period taken to first re-exacerbation. The log-rank test was utilized to determine statistical significance when comparing the curves. Multivariable Cox proportional hazards regression analyses were used to calculate the hazard ratio (HR) and 95% confidence intervals (CIs) for the first re-exacerbation associated with or without airway drugs. All data were processed and analyzed using Statistical Package for Social Sciences (SPSS), version 20.0 and Graphpad Prism 7.0.

## Result

**FIGURE 1:**
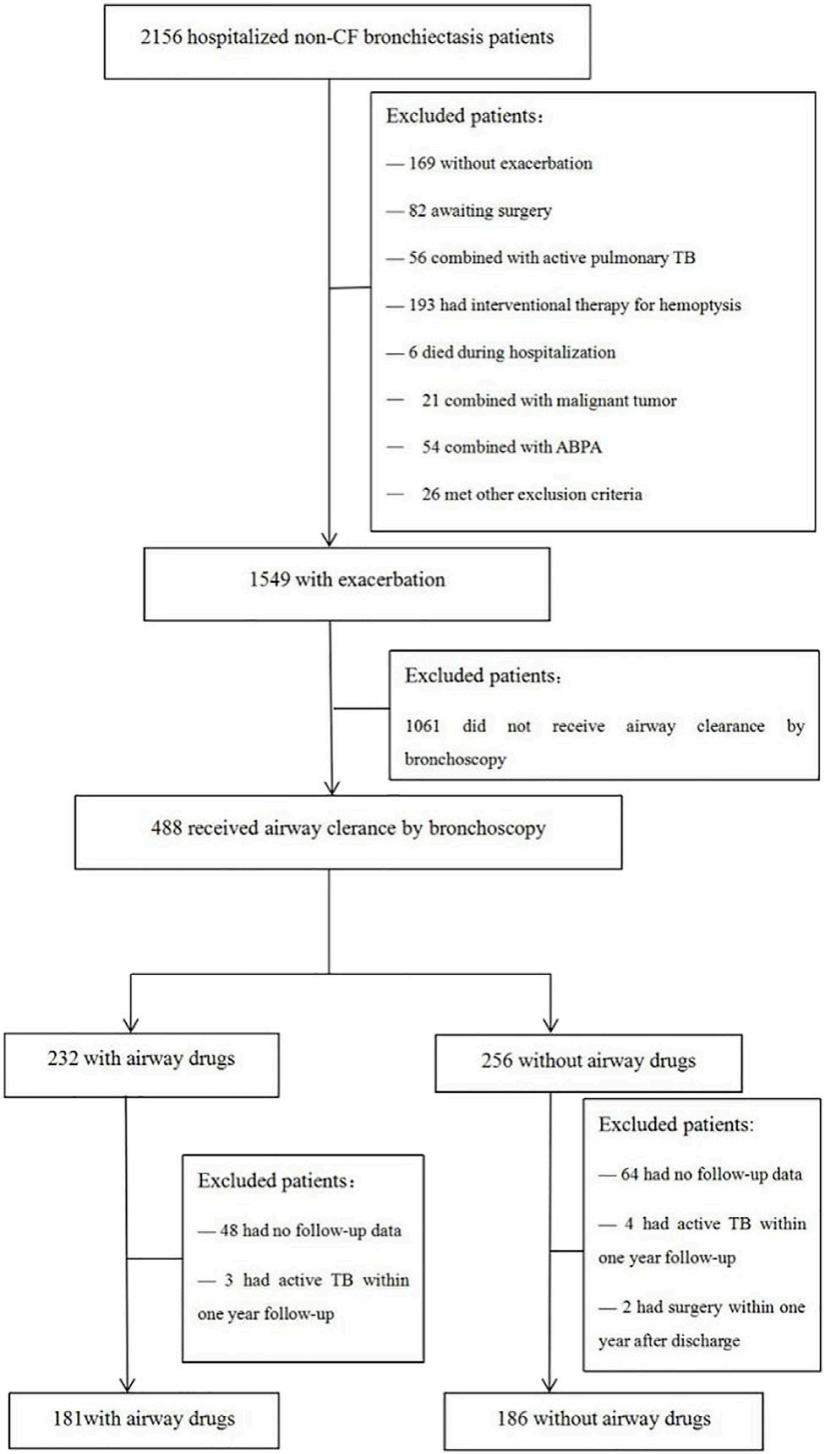
Flow chart of the study. CF, cystic fibrosis; TB, tuberculosis; ABPA, allergic bronchopulmonary aspergillosis.

A total of 2,156 hospitalized patients who were diagnosed with NCFB were enrolled in the study. 367 patients were finally included in this study, including 181 cases in the drug group and 186 cases in the control group (Figure 1).

### The Baseline Data of the Patients in the Two Groups During Exacerbation

The baseline data of the patients in the two groups during exacerbation are shown in Table 1. Although the partial pressure of oxygen (PaO_2_) in the drug group was significantly lower than that in the control group (*p* < 0.05), the mean values of the two groups were both greater than 80 mmHg, and there was no difference between the two groups in blood oxygen saturation (*p* > 0.05), which suggested that the baseline levels of PaO_2_ in the two groups were comparable. There was no significant difference between the two groups in the past medical history and comorbidities (Supplementary Table 3; all *p* > 0.05). 240 (65.40%) patients were found with secretions under the bronchoscope. At the same time, there was no significant difference between 123 cases (67.96%) in the drug group and 117 cases (62.90%) in the control group (*χ* ^2^ = 1.035, P>0.309). The mucous plug was found under the bronchoscope in 11 patients (3.00%), and no significant difference was found between the drug group {[seven cases (3.87%) and the control group [four cases (2.15%) (*χ* ^2^ = 0.930, *p* = 0.335)]}. A small amount of hemorrhage was observed in one case under the bronchoscope in each group.

**TABLE 1:** Baseline data and clinical characteristics of subjects with non-CF bronchiectasis during exacerbation.

Patient characteristic	Drug group (*n* = 181)	Control group (*n* = 186)	*P*-value
Sex (female/male)	117/64	109/77	0.234
Age (years)	54.93 ± 12.30	54.03 ± 12.68	0.490
Smoking (*n*, yes %)	31 (17.13%)	30 (16.13%)	0.797
BMI (kg/m^2^)	22.32 ± 3.16	21.72 ± 3.22	0.072
Duration of bronchiectasis (years)	14.09 ± 14.80	12.91 ± 13.37	0.921
Hb, g/L	126.37 ± 13.87	129.34 ± 15.38	0.055
Leukocyte, ×10^9^/L	6.48 ± 2.19	6.53 ± 2.23	0.866
Neutrophilic granulocyte,×10^9^/L	3.97 ± 2.05	4.09 ± 1.94	0.268
PaO_2_, mmHg	82.98 ± 14.61	86.15 ± 13.96	0.014
PaCO_2_, mmHg	40.02 ± 3.91	39.38 ± 4.78	0.169
Arterial oxygen saturation, %	95.73 ± 2.06	96.00 ± 2.64	0.064
Pulmonary function	*n* = 135	*n* = 140	—
FEV_1_, L	1.81 ± 0.80	1.86 ± 0.81	0.595
FEV_1_ predicted, %	65.15 ± 21.67	67.96 ± 23.61	0.305
FVC, L	2.54 ± 0.88	2.58 ± 0.89	0.738
FEV_1_/FVC, %	69.78 ± 12.81	70.62 ± 12.44	0.586

Data are presented as *n* (%) or mean ± SD except as otherwise noted. Hb, hematoglobin. PaO_2_, the partial pressure of oxygen. PaCO_2_, the arterial blood partial pressure of carbon dioxide. BMI, body mass index. FEV_1_, forced expiratory volume in 1s. and FVC, forced vital capacity.

There were no significant differences in the clinical symptoms in the two groups during exacerbation (Table 2; *p* > 0.05).The chest HRCT during exacerbation was compared between the two groups. There were no significant differences in all the items of the Bhalla score for all 367 patients (Supplementary Table 4; all *p* > 0.05). The detection of pathogenic bacteria during exacerbation was analyzed. The isolation rates of *PA* in the two groups (including sputum and/bronchoalveolar lavage fluid) were 28.18% (51/181) and 19.89% (37/186), respectively (*p* = 0.063). The detection rates of non-tuberculous mycobacteria were 9.94% (18/181) and 6.45% (12/186) (*p* = 0.222), and the isolation rates of other pathogenic bacteria were 6.63% (12/181) and 8.06% (15/186) (*p* = 0.599). There were no significant differences in the presence of pathogenic microorganisms between the two groups during exacerbation.

**TABLE 2:** Comparison of clinical symptom scores.

	Drug group (*n* = 181)				Control group (*n* = 186)				
	Baseline^a^	3 months later	*P-*value	*D*-value^b^	Baseline	3 months later	*P-*value	*D*-value^b^	*P-*value^c^
Cough	6.18 ± 2.00	3.03 ± 1.64^d^	<0.001	3.15 ± 1.88	6.06 ± 1.84	3.76 ± 1.70	<0.001	2.31 ± 1.70	<0.001
Expectoration	7.36 ± 2.35	4.57 ± 2.89^d^	<0.001	2.78 ± 2.53	7.21 ± 2.28	6.05 ± 2.85	<0.001	1.16 ± 2.23	<0.001
Expectoration volume	5.20 ± 2.40	2.62 ± 2.17^d^	<0.001	2.59 ± 2.04	5.44 ± 2.46	3.10 ± 2.18	<0.001	2.33 ± 1.95	0.251
Total cough score	18.75 ± 5.67	10.23 ± 5.77^d^	<0.001	8.52 ± 4.71	18.71 ± 5.35	12.90 ± 5.44	<0.001	5.81 ± 4.09	<0.001
Dyspnea	2.32 ± 2.34	1.03 ± 1.56	<0.001	1.29 ± 2.08	2.19 ± 2.19	1.26 ± 1.70	<0.001	0.94 ± 1.71	0.180
Hemoptysis	1.11 ± 2.00	0.36 ± 1.17	<0.001	0.75 ± 2.10	1.39 ± 1.98	0.47 ± 1.22	<0.001	0.92 ± 1.87	0.191
Chest pain	0.60 ± 1.43	0.23 ± 0.92	0.003	0.36 ± 1.17	0.77 ± 1.52	0.26 ± 0.95	<0.001	0.52 ± 1.47	0.131
Total symptom score	22.77 ± 7.06	11.85 ± 7.05^d^	<0.001	10.92 ± 5.89	23.06 ± 6.11	14.89 ± 6.35	<0.001	8.18 ± 4.77	<0.001

Data are presented as mean ± SD except as otherwise noted.

a There were no statistical differences in all the clinical symptom scores between the two groups during exacerbation (*p >* 0.05).

b *D*-value, difference value, the difference between baseline and 3 months later.

c The *p*-value of the *d*-value between two groups.

d There were statistical differences between the two groups 3 months later (*p* < 0.05).

### Changes in Clinical Symptoms, Chest HRCT, and Pulmonary Function

**FIGURE 2:**
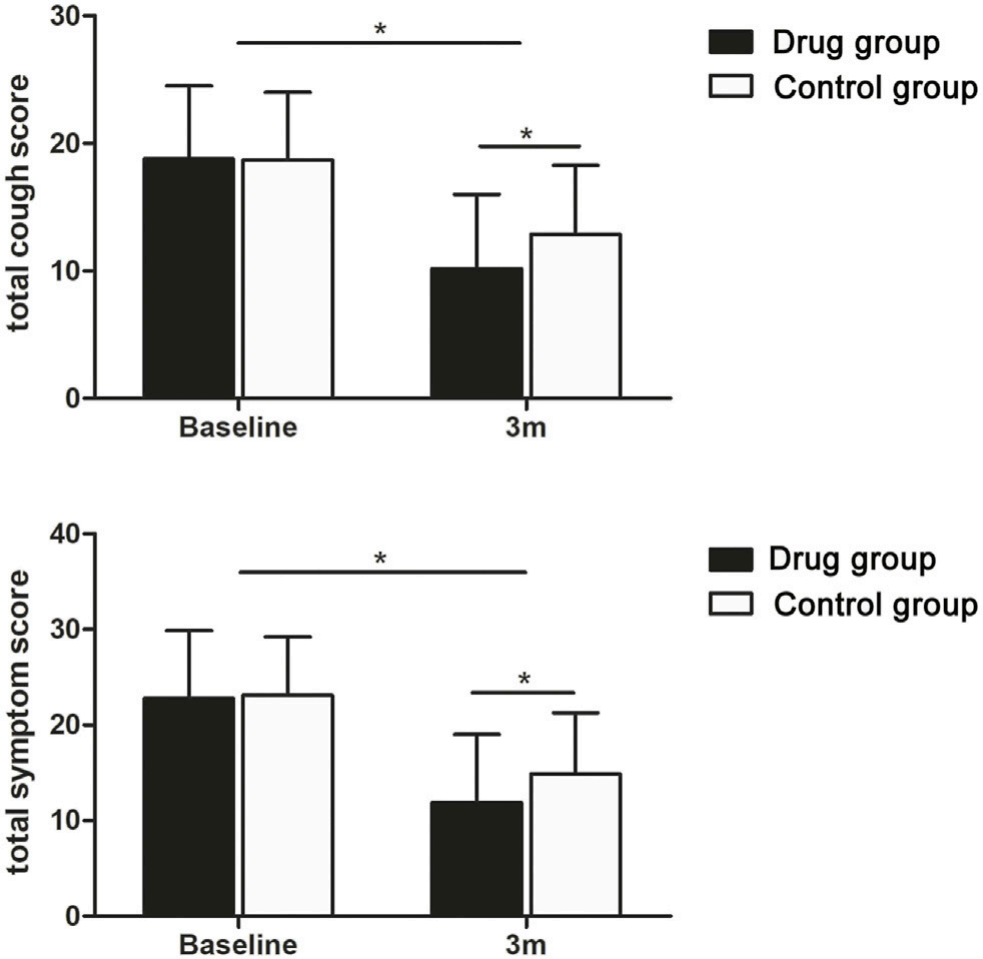
Tendency charts of the total cough score and total symptom score in the two groups three months after discharge. *p < 0.05.

**FIGURE 3:**
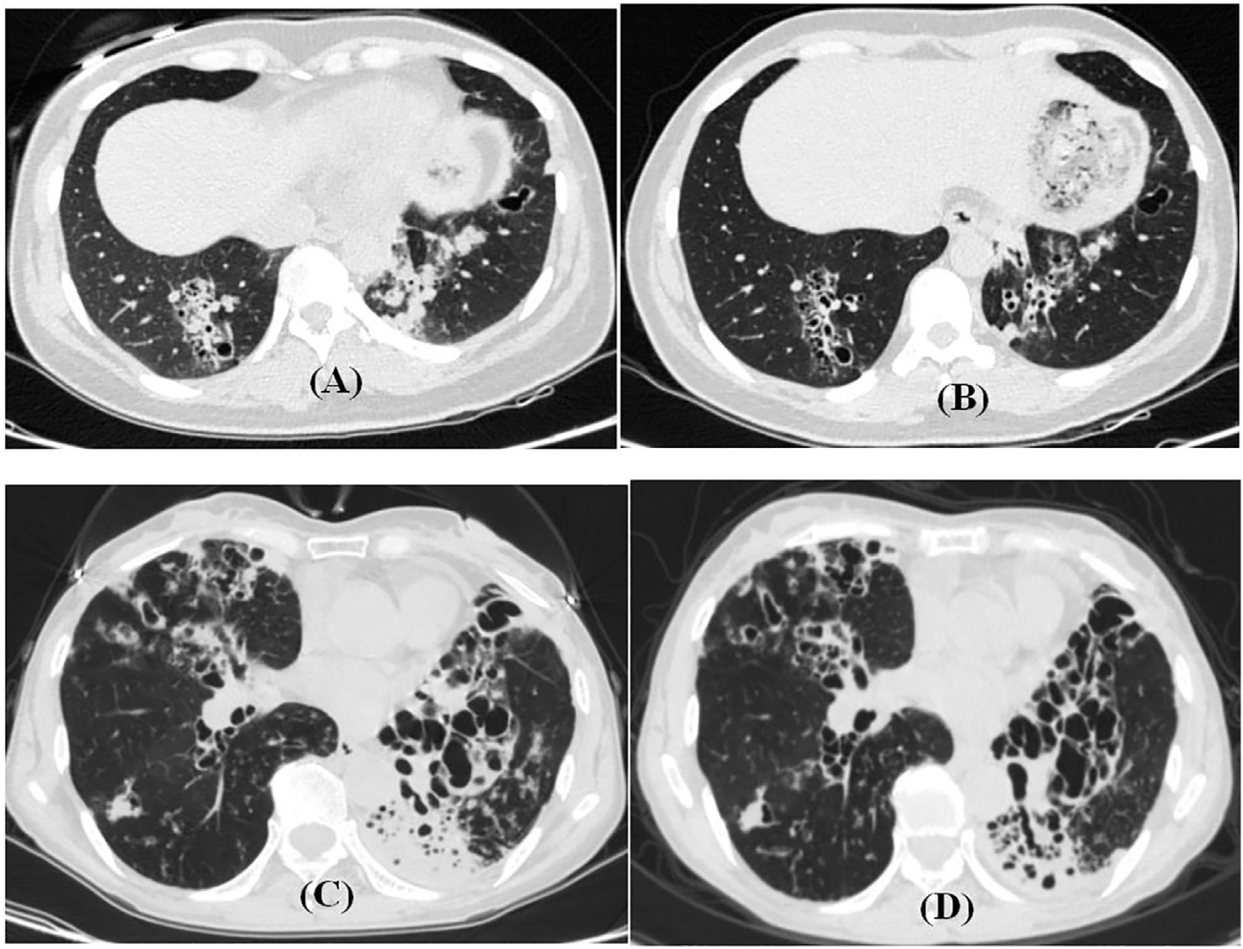
HRCT features before and after endobronchial therapy with non-CF bronchiectasis. **(A,B)** 42-year-old female patient with airway drugs. Cough and expectoration repeatedly for 4 years. **(A)** HRCT during exacerbation. **(B)** HRCT 5 months later. **(C,D)** 63-year-old female patient in the drug group. Cough and expectoration repeatedly for 40 years. **(C)**, HRCT during exacerbation. **(D)** HRCT 4 months later.

The clinical symptoms of all the patients were reassessed 3 months after discharge (all *p* < 0.05; Table 2; Figure 2), and some patients underwent chest CT (105 patients) and pulmonary function (24 patients) again within 12 months after discharge (Table 3; Figure 3 and Supplementary Table 5).

**TABLE 3:** Comparison of HRCT within 4–6 months.

	Drug group (*n* = 47)				Control group (*n* = 58)				
	Baseline	4–6 months later	*P-*value	*D*-value^a^	Baseline	4–6 months later	*P-*value	*D*-value^a^	P*-*value^b^
Severity of bronchiectasis	1.91 ± 0.75	1.81 ± 0.80	0.467	0.11 ± 0.37	2.05 ± 0.74	2.03 ± 0.75	0.903	0.02 ± 0.13	0.087
Peribronchial thickening	2.11 ± 0.67	1.64 ± 0.67	0.001	0.47 ± 0.80	2.10 ± 0.67	1.90 ± 0.69	0.104	0.21 ± 0.52	0.006
Extent of bronchiectasis (No. of BP segments)	1.60 ± 0.71^c^	1.60 ± 0.71^d^	1	0 ± 0.21	1.97 ± 0.88	1.97 ± 0.88	1	0 ± 0	1
Extent of mucous plugging (No. of BP segments)	0.98 ± 0.79^c^	0.47 ± 0.62	0.001	0.51 ± 0.59	0.52 ± 0.57	0.40 ± 0.53	0.246	0.12 ± 0.38	<0.001
Sacculations or abscesses (No. of BP segments)	0.53 ± 0.72	0.28 ± 0.58^d^	0.047	0.26 ± 0.44	0.60 ± 0.53	0.45 ± 0.54	0.105	0.16 ± 0.37	0.204
Generations of bronchial divisions involved (bronchiectasis/plugging)	2.57 ± 0.68	2.55 ± 0.75	0.982	0.02 ± 0.15	2.69 ± 0.57	2.69 ± 0.57	1	0 ± 0	0.267
No. of bullae	0.11 ± 0.52	0.11 ± 0.52	1	0 ± 0	0.21 ± 0.49	0.21 ± 0.49	1	0 ± 0	1
Emphysema (No. of BP segments)	0.34 ± 0.73	0.32 ± 0.69	0.960	0.02 ± 0.15	0.16 ± 0.41	0.16 ± 0.41	1	0 ± 0	0.267
Collapse/consolidation	1.11 ± 0.67	0.66 ± 0.73	0.002	0.45 ± 0.77	1.02 ± 0.69	0.72 ± 0.74	0.025	0.29 ± 0.59	0.133
Bhalla score	13.74 ± 3.72	15.57 ± 3.60	0.017	−1.83 ± 1.62	13.69 ± 2.89	14.48 ± 2.85	0.139	−0.79 ± 1.37	<0.001

Data are presented as mean ± SD except as otherwise noted. HRCT, high-resolution computed tomography. No, number and BP, bronchopulmonary.

a D-value, difference value, the difference between baseline and 4–6 months later.

b The *p*-value of the d-value between two groups.

c There were statistical differences between two groups during exacerbation (*p* < 0.05), the *p-*values of others were more than 0.05.

d There were statistical differences between two groups 4–6 months later (*p* < 0.05), the *p-*values of others were more than 0.05.

### The Re-Exacerbation and Antibiotic Usage

**FIGURE 4:**
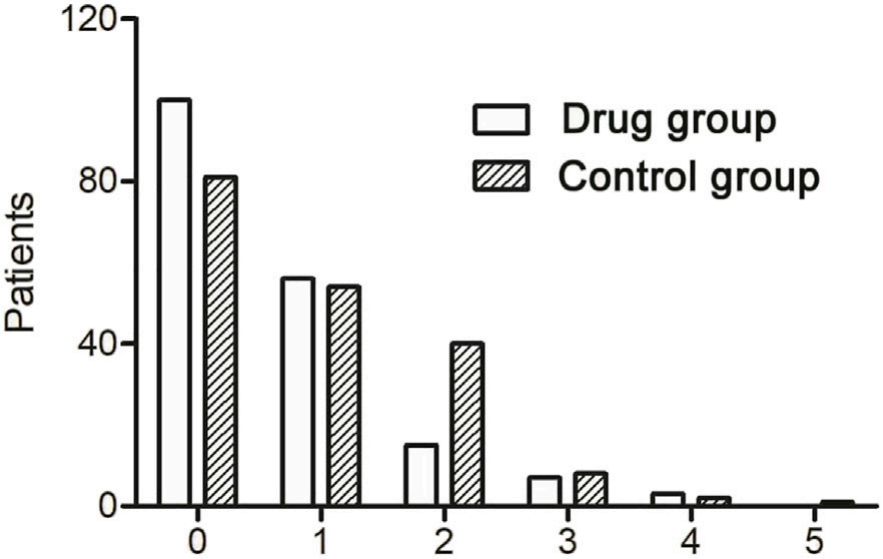
Number of re-exacerbations within 12 months in the two groups.

**FIGURE 5:**
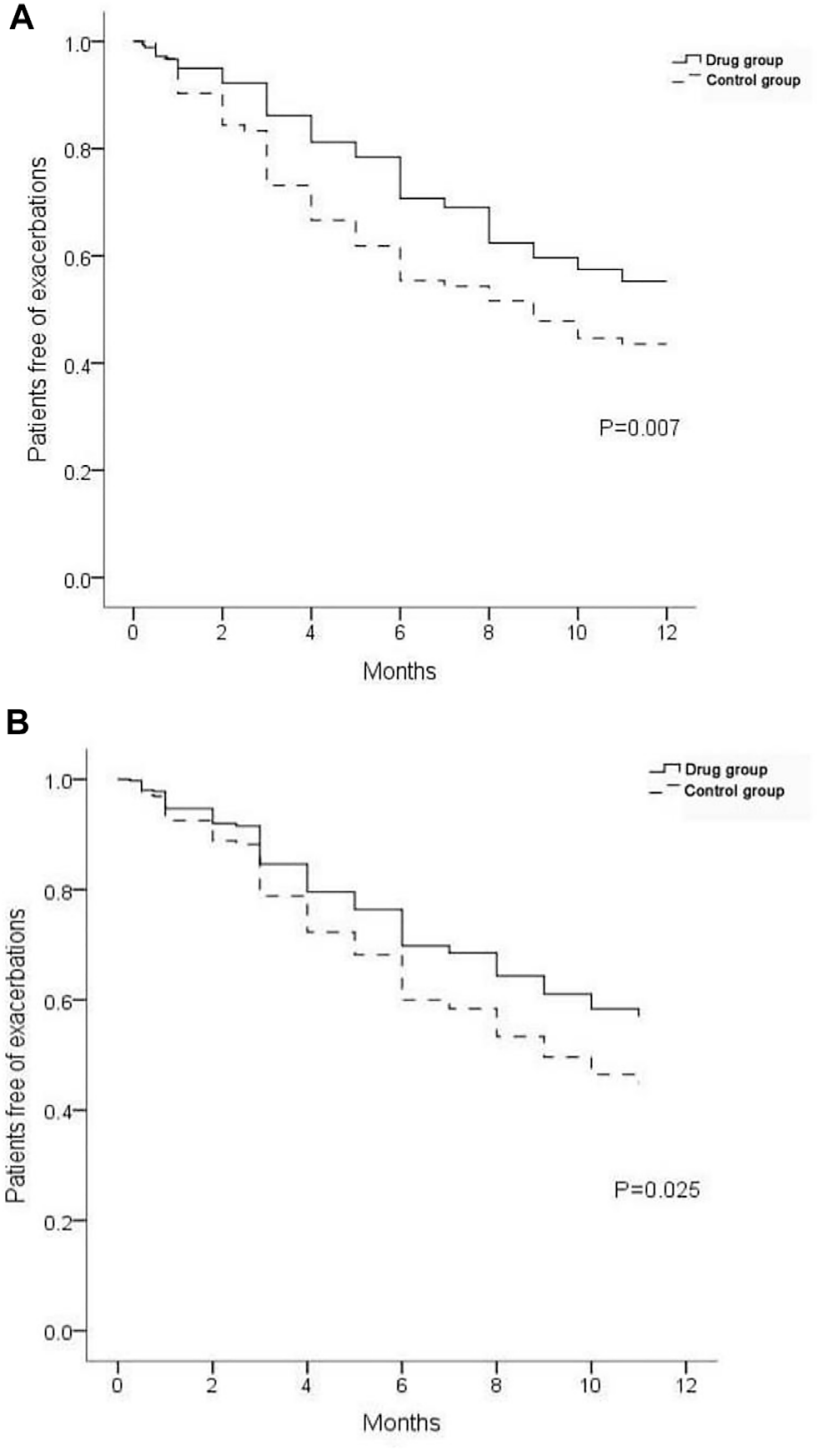
Proportion of patients free from re-exacerbations. **(A)** Result of univariate analysis. **(B)** Result of multivariate Cox regression analysis.

Within 12 months after discharge, 119 re-exacerbation events occurred in the drug group, which was significantly less (171) than those in the control group (Table 4; Figure 4). The time to first re-exacerbation was significantly prolonged in the drug group compared with the control group (6.35 ± 3.13 months versus 5.33 ± 3.00 months, *p* = 0.017), and the frequency of re-exacerbation was significantly lower in the drug group (*χ* ^2^ = 7.162, *p* = 0.007) (Figure 5A). After adjusting for age, gender, smoking history, BMI, duration of NCFB and PaO_2_, oxygen saturation, hemoglobin, total clinical symptom score, Bhalla score, and the detection rate of *PA* during an exacerbation, multiple Cox regression model results showed that the risk of the first re-exacerbation in the drug group was 70.3% that of the control group (HR = 0.703, 95% CI: 0.516–0.956, *p* = 0.025). The risk was reduced by 29.7% because of the airway drugs (Figure 5B).

**TABLE 4:** Comparisons of the number of re-exacerbations and the cumulative months of the antibiotic usage within 12 months.

	Drug group (*n* = 181)	Control group (*n* = 186)	*P*-value
No. of re-exacerbation	0.66 ± 0.91	0.92 ± 1.00	0.005
Months of oral antibiotics	1.29 ± 2.09	1.90 ± 2.33	0.001
Months of intravenous antibiotics	0.43 ± 0.79	0.65 ± 0.91	0.012

Data are presented as mean ± SD except as otherwise noted. No., number.

The number of cumulative months of the antibiotics used in the drug group was significantly lower than that in the control group within 1 year after discharge (*p* < 0.05). Moreover, 93 (51.38%) patients did not use oral antibiotics, and 127 patients (70.17%) in the drug group did not use antibiotics intravenously, which were both significantly higher than those in the control group (67, 36.02% and 110, 59.14%, *p* = 0.003 and 0.027) (Table 4).

### Adverse Events

During the bronchoscopy procedure, six patients (1.63%) altogether experienced bronchoscopy-related bleeding, including four patients in the drug group and two patients in the control group (*p* = 0.656). The bleeding stopped after local instillation of hemagglutinin or epinephrine, and there was no persistent bleeding. No other adverse events occurred during the operation procedures.

## Discussion

During the exacerbation of NCFB, the main clinical manifestations include cough, abundant and viscous sputum, and similar symptoms, all of which may affect the patients’ respiratory function and quality of life. In this study, the total cough score in patients with NCFB during exacerbation was significantly higher, the expectoration volume was increased, and the sputum was stiff. Intratracheal secretions was observed under bronchoscopy in 65.40% of the patients.

Bronchoscopy has been widely used as a routine method for diagnosing and treating airway diseases (Du Rand et al., 2011; Du Rand et al., 2013; Pizzutto et al., 2013). According to the European Respiratory Society guidelines (Polverino et al., 2017), patients with bronchiectasis should be regularly subjected to airway clearance techniques (ACT) to facilitate secretion removal and reduce cough symptoms (Lee et al., 2015). Bronchoscopy can effectively remove vast intratracheal viscous sputum and mucous plugs under direct vision, stimulate cough reflex, and facilitate expectoration of the sputum. It can also effectively promote the recruitment of airway collapse and help remove pathogenic bacteria rapidly (Kreider and Lipson, 2003). In this study, we found that after removing mucus and bronchoalveolar lavage under bronchoscopy, the cough and expectoration volume of the patients in both groups during follow-up showed significant improvement (Table 2), which suggest that mucous removal by bronchoscopy (including aspiration and lavage) is an effective method for AC in NCFB patients.

Previous studies have shown that long-term nebulized gentamicin is effective for NCFB (Murray et al., 2011). No patient had a gentamicin-resistant *PA* strain at the end of the 12 month follow-up. In this study, the local instillation of gentamicin in the NCFB lesion increased the local drug concentration and changed the living condition of the bacteria, thus directly acting as a bactericidal and bacteriostatic agent, which could avoid the inhalation-related adverse reactions.

Dexamethasone is a long-acting glucocorticoid with strong anti-inflammatory effects and can also be used as a topical medication for other diseases. For example, intratympanic dexamethasone injection can be used to treat sudden sensorineural hearing loss with no serious side effects (El Sabbagh et al., 2017). These findings support the advantages of using dexamethasone as a topical medication. Our results revealed that, based on bronchoscopy AC therapy, topical instillation of gentamicin and dexamethasone significantly improved the symptoms of cough and sputum compared to simple clearance (Table 2). The incidence of re-exacerbation was also significantly lower than that in the control group (Table 4; Figures 4, 5).

In addition to the improvement of clinical symptoms and re-exacerbation, the changes of chest HRCT showed that the local instillation of gentamicin and dexamethasone after the removal of airway mucus could alleviate the inflammation of the bronchial walls, reduce the formation of sputum plugs, and relieve the lung injury and damage through the anti-infection and anti-inflammatory mechanisms (Table 3; Figure 3).

Exacerbation implies the need for antibiotics because of the deterioration in local symptoms and/or systemic upset due to infection. In the present study, the data of re-exacerbation and antibiotic usage (Table 4) showed that endobronchial therapy with gentamicin and dexamethasone after AC by bronchoscopy could reduce exacerbation and avoid the occurrence of drug resistance and other adverse reactions caused by frequent systemic antibiotics. Furthermore, it could lessen the financial and social burden.

In this study, only six patients suffered from bronchoscopy-related bleeding. The remaining patients experienced no adverse events. The measurement of local instillation with gentamicin and dexamethasone did not bring additional adverse events. Therefore, endobronchial therapy with gentamicin and dexamethasone after AC by bronchoscopy is safe.

## Limitations

We found that endobronchial therapy with gentamicin and dexamethasone after AC by bronchoscopy is a safe and effective therapy for the exacerbation of NCFB in clinical practices. The results of this retrospective real-world study are exploratory and should be confirmed by a prospective randomized trial in the future.

## Conclusion

Endobronchial therapy after AC by bronchoscopy is a safe and effective treatment measure for the exacerbation of NCFB that can quickly and efficiently remove airway secretions under direct vision. The local application of gentamicin and dexamethasone has a direct effect on sterilization and bacteriostasis and can enhance local anti-inflammatory effects. It can also enhance the treatment outcomes, reduce and delay exacerbation, minimize the use of antibiotics, and improve the quality of life.

## Data Availability

The original contributions presented in the study are included in the article/Supplementary Material; further inquiries can be directed to the corresponding author.

## Abbreviations

NCFB, non-cystic fibrosis bronchiectasis; PA, *Pseudomonas aeruginosa*; FEV_1_, forced expiratory volume in 1 s; AC, airway clearance; HRCT, high-resolution computed tomography; ABPA, allergic bronchopulmonary aspergillosis; BMI, body mass index; SD, standard deviation; HR, hazard ratio; CIs, confidence intervals; PaO_2_, the partial pressure of oxygen; ACT, airway clearance technique; TB, tuberculosis.
