# Supplementary material for: Endobronchial Therapy With Gentamicin and Dexamethasone After Airway Clearance by Bronchoscopy in Exacerbation of Non-Cystic Fibrosis Bronchiectasis: A Real-World Observational Study
Source: Front Pharmacol. 2021 Nov 11;12:773241. doi: 10.3389/fphar.2021.773241 (PMC8632621; doi:10.3389/fphar.2021.773241)
Supplement: Supplementary file 1 [file DataSheet1.docx]

**Endobronchial therapy with gentamicin and dexamethasone after airway clearance by bronchoscope in acute exacerbation of non-cystic fibrosis bronchiectasis: A real-world observational study**

**Supplementary Table 1** Clinical symptom score scale in NCFB [ref. Torrego et al., 2006; Quittner et al., 2015; and Bai et al., 2014 in the manuscript]

| Symptom | Scores | | | |
| --- | --- | --- | --- | --- |
|  | 0 | 3 | 6 | 9 |
| Cough | Absent | Occasional short cough | Frequent cough, mild impact on daily activities | Frequent cough, serious impact on daily activities |
| Expectoration | Foamed sputum | Mucous sputum | Mucopurulent sputum | Purulent sputum |
| Expectoration volume | <10ml one day and night | 10-50ml one day and night | 51-100ml one day and night | >100ml one day and night |
| Dyspnea | Absent | Shortness of breath and chest tightness after activity | Obvious chest tightness，mild impact on daily activities | Chest tightness and shortness of breath during rest |
| Hemoptysis | Absent | Occasional bloody sputum | Sometimes bloody sputum or hemoptysis, < 10 times a day | Continuous bloody sputum or hemoptysis, > 10 times a day |
| Chest pain | Absent | Occasional chest pain | Frequent and obvious chest pain, mild impact on work | Repeated chest pain, affecting breathing, cough intolerable |

Total cough score is the sum of cough, expectoration and expectoration volume. Total symptom score is the sum of all the six symptom scores.

**Supplementary Table 2** Bhalla scoring system [ref. Bhalla et al., 1991 and Oikonomou et al., 2008 in the manuscript]

| Category | Scores | | | |
| --- | --- | --- | --- | --- |
|  | 0 | 1 | 2 | 3 |
| Severity of bronchiectasis | Absent | Mild (luminal diameter slightly greater than the diameter of an adjacent blood vessel) | Moderate (lumen 2–3 times the diameter of the vessel) | Severe (lumen >3 times diameter of vessel) |
| Peribronchial thickening | Absent | Mild (wall thickness equal to the diameter of an adjacent blood vessel) | Moderate (wall thickness greater than and up to twice the diameter of adjacent vessel) | Severe (wall thickness > 2 times the diameter of adjacent vessel) |
| The extent of bronchiectasis (no. of BP segments) | Absent | 1-5 | 6-9 | ＞9 |
| The extent of mucous plugging (no. of BP segments) | Absent | 1-5 | 6-9 | ＞9 |
| Sacculations or abscesses (no. of BP segments) | Absent | 1-5 | 6-9 | ＞9 |
| Generations of bronchial divisions involved (bronchiectasis/plugging) | Absent | Up to 4th generation | Up to 5th generation | Up to 6th generation and distal |
| No. of bullae | Absent | Unilateral (not >4) | Bilateral (not >4) | ＞4 |
| Emphysema (no. of BP segments) | Absent | 1-5 | ＞5 | - |
| Collapse/consolidation | Absent | Subsegmental | Segmental/lobar | - |

BP: bronchopulmonary. The Bhalla score is 25 minus the above nine scores. The higher the score of Bhalla, the lighter the degree.

**Supplementary Table 3** The past medical history and comorbidity of patients

|  | Drug group (n=181) | Control group (n=186) | *P*-value |
| --- | --- | --- | --- |
| Past history |  |  |  |
| Tuberculosis | 15(8.29%) | 23(12.37%) | 0.200 |
| Other infectious diseases | 7(3.87%) | 5(2.69%) | 0.525 |
| History of exposure to poisons or dust | 7(3.87%) | 2(1.08%) | 0.164 |
| Comorbidity |  |  |  |
| COPD | 29(16.02%) | 42(22.58%) | 0.112 |
| Asthma | 10(5.52%) | 15(8.06%) | 0.334 |
| Rhinitis and nasosinusitis | 20(11.05%) | 14(7.53%) | 0.245 |
| Connective tissue disease | 6(3.31%) | 5(2.69%) | 0.725 |
| Inherited disease | 1(0.55%) | 2(1.08%) | 1 |
| Respiratory failure | 8(4.42%) | 6(3.23%) | 0.550 |
| Hypertension | 26(14.36%) | 27(14.52%) | 0.967 |
| Diabetes | 11(6.08%) | 14(7.53%) | 0.582 |
| Coronary heart diseases | 3(1.66%) | 1(0.54%) | 0.596 |
| Gastroesophageal disease | 8(4.42%) | 10(5.38%) | 0.671 |

Data are presented as n (%) except as otherwise noted. COPD, chronic obstructive pulmonary disease.

**Supplementary Table 4** HRCT during exacerbation

|  | Drug group（n=181） | Control group（n=186） | *P*-Value |
| --- | --- | --- | --- |
| Severity of bronchiectasis | 1.84±0.82 | 1.84±0.83 | 0.920 |
| Peribronchial thickening | 2.14±0.78 | 2.16±0.75 | 0.819 |
| Extent of bronchiectasis  (No. of BP segments) | 1.82±0.80 | 1.81±0.79 | 0.895 |
| The extent of mucous plugging  (No. of BP segments) | 0.70±0.74 | 0.56±0.65 | 0.093 |
| Sacculations or abscesses  (No. of BP segments) | 0.38±0.57 | 0.35±0.50 | 0.985 |
| Generations of bronchial  divisions involved  (bronchiectasis/plugging) | 2.64±0.60 | 2.76±0.46 | 0.073 |
| No. of bullae | 0.08±0.35 | 0.12±0.38 | 0.119 |
| Emphysema  (No. of BP segments) | 0.35±0.68 | 0.38±0.67 | 0.443 |
| Collapse/consolidation | 0.82±0.70 | 0.77±0.69 | 0.507 |
| Bhalla total score | 14.23±3.62 | 14.17±3.17 | 0.699 |

Data are presented as mean±SD except as otherwise noted. HRCT, High Resolution Computed Tomography. No., number. BP, bronchopulmonary.

**Supplementary Table 5** Pulmonary function during exacerbation (Baseline) and within 12 months

|  | Drug group (n=14） | | | Control group（n=10） | | |
| --- | --- | --- | --- | --- | --- | --- |
|  | Baseline | Within 12 months | *P*-value | Baseline | Within 12 months | *P*-value |
| FEV_1_，L | 1.83±0.86 | 1.83±0.83 | 0.910 | 1.22±0.38 | 1.40±0.46 | 0.307 |
| FEV_1_ % predicted | 62.14±26.02 | 63.47±25.12 | 0.946 | 44.32±13.20 | 51.28±14.89 | 0.186 |
| FVC，L | 2.57±0.88 | 2.63±0.88 | 0.804 | 2.17±0.36 | 2.39±0.53 | 0.345 |
| FVC % predicted | 71.74±19.14 | 75.21±19.67 | 0.635 | 63.81±9.24 | 70.68±8.95 | 0.140 |
| FEV_1_/FVC，% | 68.11±16.02 | 66.75±15.06 | 0.946 | 55.55±10.07 | 58.05±10.78 | 0.496 |

Data are presented as mean±SD except as otherwise noted. FEV_1_, forced expiratory volume in 1s. FVC, forced vital capacity.
